# 2420: Loss of eptB decreases systemic inflammation during *Salmonella* infection and allows for evasion of the host immune response

**DOI:** 10.1017/cts.2017.47

**Published:** 2018-05-10

**Authors:** Lillian F. Zhang, Fabian Rivera-Chavez, Hirotaka Hiyoshi, Andreas J. Baumler

## Abstract

OBJECTIVES/SPECIFIC AIMS: Our long-term goal is to elucidate the molecular mechanisms and virulence factors that control the differential presentation of infection with Salmonella typhimurium and Salmonella typhi. The objectives of this study are to study the mechanisms that enable S. typhi to trigger a decreased inflammatory response in comparison with S. typhimurium and evade detection by the immune system, leading to the development of asymptomatic chronic carriage of S. typhi. METHODS/STUDY POPULATION: A loss of function eptB mutant S. typhimurium strain was generated. Lipopolysaccharide (LPS) was isolated from wild-type and eptB mutant S. typhimurium and wild-type S. typhi. Binding of LPS to recombinant intelectin was tested by dot blot and enzyme-linked immunosorbant assay (ELISA). C57BL/6 mice were infected with wild-type or eptB mutant S. typhimurium by oral gavage and inflammatory cytokines in the spleen, liver, and Peyer’s patches were measured by qPCR. RESULTS/ANTICIPATED RESULTS: LPS isolated from wild-type S. typhimurium is not bound by intelectin, a protein that has been proposed to function in innate immunity and that is known to be able to bind certain moieties within LPS. Conversely, LPS isolated from eptB mutant S. typhimurium and wild-type S. typhi, which lacks a functional eptB, is bound by intelectin. Mice infected with an eptB mutant S. typhimurium exhibit decreased expression of inflammatory cytokines in the spleen compared to mice infected with the wild type S. typhimurium, suggesting that loss of eptB function allows a nontyphoidal Salmonella serovar to mimic the stealth phenotype of typhoidal serovars. Together, these results suggest that loss of eptB function allows intelectin to bind to and detoxify Salmonella LPS, leading to decreased systemic inflammation during infection. DISCUSSION/SIGNIFICANCE OF IMPACT: These results have broad implications for how pathogens such as S. typhimurium induce systemic shock during infection and may also help to explain a mechanism for how S. typhi is able to evade immune detection and enhance dissemination to systemic sites, leading to development of the asymptomatic chronic carrier state. Further investigation of this novel virulence mechanism will mark a decisive step forward in understanding the mechanisms underlying the differential pathogenesis of S. typhimurium-induced gastroenteritis and S. typhi-induced typhoid fever. Additionally, these results contribute to our understanding of the interactions between host and pathogen in affecting disease presentation, which will have wide appeal among researchers interested in microbial pathogenesis and the contribution of host-pathogen interactions to health and disease.

